# Impact of dosimetric factors on long-term percutaneous enteral gastrostomy (PEG) tube dependence in head and neck cancer patients after (chemo)radiotherapy—results from a prospective randomized trial

**DOI:** 10.1007/s00066-022-01992-5

**Published:** 2022-08-25

**Authors:** Anastassia Löser, Maximilian Grohmann, Anna Finger, Franziska Greinert, Linda Krause, Isabel Molwitz, Andreas Krüll, Cordula Petersen

**Affiliations:** 1 Department of Radiotherapy and Radiation Oncology, University Medical Center Hamburg-Eppendorf, Outpatient Center of the UKE GmbH, Martinistr. 52, 20246 Hamburg, Germany; 2 Department of Oncology, Hematology and Bone Marrow Transplantation with the Section Pneumology, Center for Oncology, University Medical Center Hamburg-Eppendorf, Martinistr. 52, 20246 Hamburg, Germany; 3 Institute of Medical Biometry and Epidemiology, University Medical Center Hamburg-Eppendorf, Martinistr. 52, 20246 Hamburg, Germany; 4 Department of Diagnostic and Interventional Radiology and Nuclear Medicine, University Medical Center Hamburg-Eppendorf, Martinistr. 52, 20246 Hamburg, Germany; 5 Department of Radiotherapy and Radiation Oncology, University Medical Center Hamburg-Eppendorf, Martinistr. 52, 20246 Hamburg, Germany

**Keywords:** Larynx, Inferior constrictor muscle, PEG tube, DVH parameters, Toxicity

## Abstract

**Purpose/objective:**

To analyze dose–volume histogram (DVH)-derived data on the exposure of organs at risk with impact on long-term percutaneous enteral gastrostomy (PEG) tube dependence in head and neck cancer patients at 6 and 12 months after definitive or adjuvant (chemo)radiotherapy.

**Materials and methods:**

Sixty-one patients were prospectively treated with (chemo)radiotherapy. Prophylactic or reactive gastrostomy tube placement was performed in 41 (67.2%) patients. Dose–volume histogram parameters were obtained for the swallowing apparatus.

**Results:**

Median follow-up time was 25 (2–34) months. Overall survival was shorter in patients with inlying PEG tubes at 6 and 12 months (log rank *p* = 0.038 and *p* = 0.017) after therapy completion. The estimated median time of tube dependency was 6 (95% confidence interval: 2–14) months. After 6 months, 46.5% of patients were tube dependent. After 12 months, this estimated proportion fell to 31.5%. For both time points, the volume to the larynx (in %) receiving at least 50 Gy (larynx V50Gy) exceeding 53% was predictive for long-term tube feeding (6 months: *p* = 0.041 and 12 months: *p* = 0.042) being an independent predictor during multivariable analysis. There was no clinical feature influencing tube dependence after 12 months.

**Conclusion:**

Long-term gastrostomy dependence was found to be strongly associated with an exposure of laryngeal structures (specifically, V50Gy ≥ 53%) during radiotherapy. Consequently, the avoidance of supraglottic as well as glottic structures is warranted.

## Introduction

Curative treatment options for head and neck cancer (HNC) patients include surgery, radiotherapy and/or chemotherapy, or a combination of these options depending on the oncological risk profile. Although intensity-modulated radiotherapy (IMRT) significantly improved the sparing of organs at risk (OAR) and thus reduced radiotherapy-associated side effects, patients are still confronted with adverse effects like dysphagia or mucositis. In particular, the combination of therapy modalities, e.g., chemoradiotherapy, even favors the worsening of swallowing difficulties and, thus, increases weight loss and malnutrition. To compensate for these therapy-related side effects, a percutaneous enteral gastrostomy (PEG) tube is inserted in clinical practice if a swallowing deficit is expected (prophylactic) or already exists (reactive).

Doses to the swallowing structures, like the OAR larynx and pharyngeal constrictors, seem to be associated with long-term swallowing complications and/or long-term PEG tube dependence [1–4]. Long-term PEG tube need is linked to poorer overall survival in HNC patients [5]. According to Caudell et al., a mean dose (Dmean) to the larynx exceeding 41 Gy and a laryngeal volume of more than 24% receiving 60 Gy (V60Gy > 24%), as well as V60Gy > 12% to the inferior pharyngeal constrictor correlated with PEG tube dependence and aspiration. Also, these authors reported that V65Gy > 33% to the superior and V65Gy > 75% to the middle pharyngeal constrictor was linked to dilation-worthy strictures [1]. Schwartz et al. prospectively investigated radiation-induced long-term dysphagia in 31 patients with squamous cell carcinoma of the oropharynx. In addition to the high superior pharyngeal constrictor (V55Gy < 80% and V65Gy < 30%), they found the dose to the anterior oral cavity (V30Gy < 65% and V35Gy < 35%) being predictive for swallowing complications [6].

Data on the association between the applied radiation dose to the swallowing muscles and long-term PEG dependence of HNC patients are still limited and are mostly derived from retrospective analyses. This prospective analysis focuses on the radiation doses delivered to the swallowing muscles and the larynx as well as their impact on long-term PEG dependence at 6 and at 12 months after therapy completion.

## Materials and methods

### Study design and patient selection

Our presented data were generated prospectively at the Department of Radiotherapy and Radiation Oncology, University Medical-Center Hamburg-Eppendorf within the framework of the HEADNUT trial (*head* and neck cancer patients under (chemo-)radiotherapy undergoing *nut*ritional intervention) [7]. This trial was approved by the local ethics committee (PV5818) and registered within the German Clinical Trials Register (DRKS00016862). HEADNUT patients were randomized 1:1 into a control and intervention group, the latter receiving nutritional counseling every 2 weeks during therapy [7]. This work focuses on the correlation between the radiation dose delivered to the swallowing muscles and long-term PEG dependence at 6 and 12 months after (chemo)radiotherapy. Our follow-up period lasted until January 2022.

Between October 2018 and October 2020, 220 patients were prospectively screened for their study eligibility: 142 patients did not meet the inclusion criteria due to a palliative treatment intent, nonsquamous cell HNC, denial of radiotherapy or study participation, an inlying pacemaker as a relative contraindication to bioelectrical impedance analysis (BIA; applied within the HEADNUT trial to assess body composition), nasopharyngeal carcinoma, or a known solid tumor disease within the last 15 years, and other reasons. Nine patients could not be randomized due to a recruitment stop during the Corona pandemic in Germany. Of the remaining 69 patients, 8 patients did not receive the allocated treatment due to various reasons. Finally, 61 were treated in the HEADNUT trial [7]. All included patients were at least 18 years old, had a Karnofsky performance status ≥ 60%, and suffered from squamous cell carcinoma of the oropharynx, oral cavity, hypopharynx, larynx, or the salivary glands (see [7] for CONSORT diagram). Written informed consent was obtained from all patients before entering this study.

### Treatment and PEG tube placement

Data on our therapy procedure have previously been published [7–9]. Briefly, patients were referred to our department for either definitive or adjuvant radiotherapy (IMRT with 1.7–2.0 Gy, 5 times/week, to cumulative doses ranging from 60–70.4 Gy [10, 11]) alone or combined chemoradiotherapy with mainly cisplatin (100 mg/m^2^ 3‑weekly or 40 mg/m^2^ weekly) or with 5‑fluorouracil (5-FU)/mitomycin C (MMC) (600 mg/m^2^ on days 1–5 and 10 mg/m^2^ on days 5 and 36, respectively).

PEG tube placement was performed at the Department of Interventional Radiology. Pretreatment reasons for inserting a PEG tube were definitive chemoradiotherapy, bilateral radiotherapy to the neck, and the patient’s pretherapeutic clinical condition (e.g., preexisting dysphagia). However, the decision of prophylactic PEG tube placement was left to the attending physician. Under ongoing therapy, reactive tube placement mainly occurred due to therapy-related side effects leading to dysphagia with worsening nutritional status. Regardless of the presence of a PEG tube, all patients were encouraged to ingest food orally. The degree of severity for dysphagia was classified according to the National Cancer Institute Common Terminology Criteria for Adverse Events (CTCAE, version 5) [12].

### Contouring of swallowing muscles

**Fig. 1:**
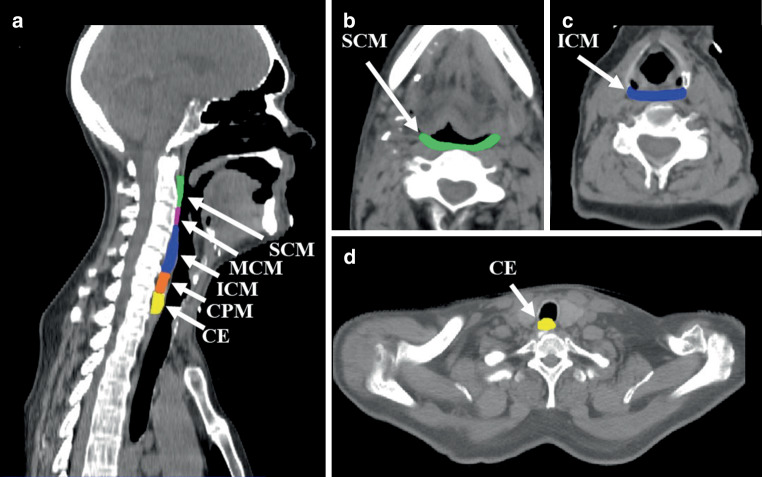
Defined muscular areas of the swallowing apparatus on **a** sagittal and **b**–**d** axial unenhanced CT slices. **a** gives an overview of the substructures of the swallowing apparatus, while **b–d** show selected swallowing structures. *SCM* Superior pharyngeal constrictor muscle, *MCM* Middle pharyngeal constrictor muscle, *ICM* Inferior pharyngeal constrictor muscle, *CPM* Cricopharyngeal muscle, *CE* Cervical esophagus

The superior (SCM), middle (MCM), and inferior pharyngeal constrictor muscle (ICM), the cricopharyngeal muscle (CPM; sometimes referred to as being a part of the ICM), and the cranial 1 cm part of the cervical esophagus, known as the esophageal inlet, are considered to be of pre-eminent importance for swallowing [13]. Contouring of the aforementioned swallowing muscles was performed according to anatomical landmarks (SCM: mid C2 to upper C3; MCM: upper C3 to upper C4/caudal part of the corpus of the hyoid bone; ICM: upper C4 to mid C6; CPM: mid C6 till esophageal junction) on 3‑mm axial planning CT slices (Somatom, Siemens Healthcare, Forchheim, Germany) using Eclipse (v15.1, Varian Medical Systems, Inc., Palo Alto, CA, USA) by the same investigator (Fig. 1; [13]). In addition, we contoured the larynx as one structure reaching from the vocal cords to the epiglottis [1]. For a better anatomical delineation additional contrast-enhanced CT scans were obtained. As part of the prospective therapy planning, the muscular swallowing apparatus was contoured as one structure. For a more precise differentiation of the individual structures (SCM, MCM, ICM, CPM, and the esophageal inlet), retrospective postcontouring was carried out for this analysis.

In accordance with previously published studies, we extracted the dose–volume histogram parameters Dmax (maximum dose; Gy), Dmean (mean dose; Gy), V40Gy (%), V50Gy (%), V60Gy (%), and V65Gy (%) [1, 14]. VxGy (%) is defined as the organ volume (%) that received ≥ x Gy.

### Follow-up

During ongoing therapy, all patients were seen by a radiation oncologist being part of a permanent study team every 2 weeks. Approximately 6–8 weeks after the completion of (chemo)radiotherapy, the first posttherapeutic follow-up session took place. Afterwards, follow-up occurred semiannually. During each follow-up session, the patient’s clinical condition, the nutritional status (by means of weight loss and BIA), and the need for maintaining the PEG tube were re-evaluated.

In patients with a stable weight course via oral feeding without other complications like dysphagia or aspiration, removal of the PEG tube was initiated.

Since the time interval of long-term PEG use is not uniformly defined among previously published studies, we analyzed two different time points, namely at 6 months and at 12 months after therapy completion [1, 14].

### Statistical analysis

The endpoint of this analysis was the removal of gastrostomy tube. To analyze the incidence of long-term PEG dependency, all patients whose follow-up was less than 6 and 12 months (after the end of radiotherapy) were excluded.

To test for normal distribution, the Kolmogorov–Smirnov test was applied. For descriptive statistics we indicated the median with the corresponding range (nonnormally distributed data) or the mean with standard deviation (± SD) (normal distribution). The Fisher’s exact test was used to compare results within a 2 × 2 contingency table. Otherwise, to examine categorical variables with more than two levels, χ^2^ test was chosen. The Mann–Whitney U‑test was utilized to evaluate differences in one dependent variable within two independent groups (Tables 1 and 2). This test was also used to evaluate the gross target volume (GTV) as another possible influencing factor on tube dependence after 6 and 12 months (GTV volume, Dmax, Dmean, V40Gy, V50Gy, V60Gy, and V65Gy [in %]).

**Table 1:** Patient characteristics

	Definitive		Adjuvant		–
	Number	%	Number	%	*p*-value
	*n* = 29	47.5	*n* = 32	52.5	–
*Pretreatment dysphagia*					0.59^a^
None	21	72.4	21	65.6	–
Present	8	27.6	11	34.4	
*Prophylactic or reactive tube placement (n* *=* *41)*	*19*	*65.5*	*22*	*68.8*	1^a^
*Treatment group*					0.31^a^
Intervention group (arm A)	18	62.1	15	46.9	–
Control group (arm B)	11	37.9	17	53.1	
*Age in years, median (range)*	63 (20–82)	–	63 (50–89)	–	0.63^b^
*Male gender*	23	79.3	21	65.6	0.27^a^
*Karnofsky performance status ≥* *80%*	28	96.6	25	78.1	0.06^b^
*Primary site*					0.06^c^
Oropharynx	21	72.4	17	53.1	–
Oral cavity	1	3.4	9	28.1	
Hypopharynx	1	3.4	3	9.4	
Larynx	4	13.8	1	3.1	
Other	2	6.9	2	6.3	
*UICC classification, 8th edition*					0.45^c^
I	10	34.5	9	28.1	–
II	5	17.2	6	18.8	
III	7	24.1	4	12.5	
IV	7	24.1	13	40.6	
*T‑classification, 8th edition*					0.51^c^
T1	5	17.2	4	12.5	–
T2	11	37.9	13	40.6	
T3	2	6.9	6	18.8	
T4	11	37.9	9	28.1	
*N‑classification, 8th edition*					0.66^c^
N0	4	13.8	7	21.9	–
N1	11	37.9	14	43.8	
N2	12	41.4	8	25	
N3	2	6.9	3	9.4	
*Concurrent chemotherapy*					*0.004*^a^
Yes	22	75.9	13	40.6	–
No	6	20.7	19	59.4	
Cetuximab	1	3.4	0	0	
*PTV high dose (Gy)*					*<* *0.001*^c^
≥ 70	27	93.1	2	6.3	–
66	2	6.9	17	53.1	
60	0	0	13	40.6	
*Baseline smoking status*					0.48^c^
Never smoked	11	37.9	10	31.3	–
Active smoker	9	31	6	18.8	
Ex-smoker	4	13.8	7	21.9	
Unknown	5	17.2	9	28.1	

*PTV* Planning target volume, *UICC* Union Internationale Contre le Cancer

^a^ Fisher’s exact test

^b^ Man–Whitney U‑test

^c^ Chi-square (χ^2^) test

**Table 2:** Univariable and multivariable analysis on relevant dose–volume histogram (DVH) parameters for gastrostomy tube dependence in all patients after prophylactic or reactive tube placement

***Univariable analysis***					
Gastrostomy tube dependence after 6 months, median (range)					
	Larynx V50Gy (%)			Karnofsky status (%)	Tumor location
≥ 6 months	73.9 (0–100)			80 (60–100)	–
< 6 months	16.4 (0–53)			90 (70–100)	–
*p*-value	0.041			0.02^a^	0.04^b^
Gastrostomy tube dependence after 12 months, median (range)					
	Larynx V50Gy (%)	ICM V60Gy (%)		ICM V65Gy (%)	–
≥ 12 months	86.1 (0.5–100)	67.6 (0.1–100)		41.6 (0–99.1)	–
< 12 months	15.3 (0–100)	24.2 (0–93.2)		2.2 (0–85.3)	–
*p*-value	0.042	0.047		0.042	–
***Multivariable logistic regression model***					
Independent variable	Logistic regression coefficient/constant	Odds ratio (95% CI)	SE	*p*-value	–
Larynx V50Gy (%)	0.035/–2.14	1.04 (1.01–1.06)	0.01	0.01	–

VxGy (percentage [%] of volume) is defined as the percentage of organ volume (in %) that received at least x Gy. The following parameters were entered into the multivariable regression model: Larynx V50Gy (%), ICM V60Gy (%) and ICM V65Gy (%) (model’s area under the curve [AUC] = 0.74)

*DVH* Dose-volume histogram, *ICM* Inferior pharyngeal constrictor muscle, *SE* Standard error, *95% CI* 95% confidence interval

^a^ Mann–Whitney U‑test

^b^ Chi-square (χ^2^) test

When there were more than two independent samples, the Kruskal–Wallis test was used. In addition, a Pearson (normal distribution) or Spearman (non-normally distributed data) correlation coefficient was determined (correlation of DVH parameters with the swallowing structures). Apart from the GTV, CPM Dmean and larynx Dmean, all other dosimetric parameters showed normal distribution.

We assumed that the following clinical factors may influence long-term PEG tube dependence at 6 and 12 months: Tumor location and stage, Karnofsky performance status, the presence of pretherapeutic dysphagia, and the performance of radiotherapy alone versus concomitant chemoradiotherapy. The χ^2^ test or Fisher’s exact test was used to test for possible differences. Expecting that upfront surgery may have affected the patients’ swallowing ability, the patient collective was also studied separately for definitive versus adjuvant therapy (Table 1).

To estimate patients’ survival and the course of PEG tube removal after the end of radiotherapy, the Kaplan–Meier method was used. Corresponding effects were investigated using the log rank test. Estimated medians were presented together with the corresponding 95% confidence intervals (CI). After performing univariable Kaplan–Meier analysis, all identified relevant DVH parameters for tube dependence after 12 months (larynx V50Gy, ICM V60Gy, and ICM V65Gy [all in %]) were introduced into a multivariable regression model (by backward stepwise selection) as continuous variables (MedCalc). Kaplan–Meier method (including the presented Kaplan–Meier curves) was conducted with MedCalc (version 19.6, MedCalc Software Ltd, Ostend, Belgium). Youden indices from receiver operating characteristics (ROC) curves were used to define thresholds for relevant DVH parameters (MedCalc). All other calculations were carried out with SPSS (version 25.0, IBM Corp., Armonk, NY, USA). In this exploratory study, *p*-values are used as descriptive summary measures and were not adjusted for multiple testing.

## Results

### Patient characteristics

Our previous works did not show any differences between the control and intervention group regarding general patient features and different oncological endpoints [7, 8]. Accordingly, we separated our patient population into those treated with definitive versus adjuvant combined chemoradiotherapy or radiotherapy alone: In both groups (definitive vs. adjuvant setting), 21 patients (34.4% each) denied the presence of pretreatment swallowing disorders. At baseline, CTCAE grade 1/2 dysphagia was present in 8 patients (13.1%) in the definitive and in 11 patients (18%) in the adjuvant setting (*p* = 0.8).

As previously described, a prophylactic gastrostomy tube was present in 33 patients (54.1%), while reactive tube placement was performed in 6 patients (9.8%) [7], without differences between the control and intervention group (*p* = 0.79). In a 7th patient, the PEG tube had to be removed during ongoing therapy due an infection of the abdominal cutaneous entry site. PEG tube re-insertion was refused by this patient. In another patient, the PEG tube was inserted during ongoing therapy and removed 1 day before the end of radiotherapy at the patient’s request. In summary, there were 41 patients (67.2%) who either had undergone prophylactic or reactive tube placement. None of our patients received postradiotherapy neck dissection. Patient characteristics are summarized in Table 1.

### Patient outcome and incidence of long-term PEG tube dependence

Median overall follow-up was 25 (2–34) months. Within this follow-up interval, 9 patients (14.8%) died. Four of them (6.6%) passed away due to disease progression (local recurrence or distant metastases; cancer-related) and in 5 of them (8.2%) the cause of death was either unknown or not cancer-related. Overall survival was not influenced by prophylactic or reactive tube placement at baseline (log rank *p* = 0.13). However, overall survival was shorter in patients with long-term PEG use at 6 months and 12 months after (chemo)radiotherapy (log rank *p* = 0.038 and *p* = 0.017). None of our patients died from complications after PEG tube placement.

**Fig. 2:**
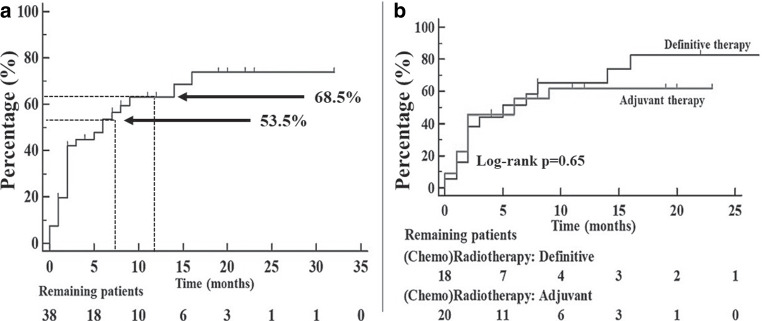
Kaplan–Meier curves on the removal of gastrostomy (PEG). All patients whose follow-up was less than 6 and 12 months were excluded. After 6 months, the estimated proportion of PEG tube dependent patients equaled 46.5%, and after 12 months, it fell to 31.5%. Kaplan–Meier curve in **a** refers to the tube removal in all patients with either prophylactic or reactive tube placement (3 patients already censored at the beginning). The time points at 6 and 12 months are marked. In **b**, patients were grouped according to their therapy setting (definitive vs. adjuvant)

Estimated median time of tube dependency was 6 (95% CI 2–14) months. After 6 months, the estimated proportion of PEG tube dependent patients equaled 46.5%, and after 12 months, it fell to 31.5% (Fig. 2a).

### Association between clinical features and long-term PEG tube dependency

There were fewer patients with a baseline Karnofsky performance status of ≥ 80% who still needed their PEG tube after 6 months (*p* = 0.02). Tumor location also played an important role. Especially patients with tumor primaries in the oropharynx and oral cavity were still PEG tube dependent after 6 months (*p* = 0.04). After 12 months, none of the tested clinical factors were associated with long-term PEG tube dependence (Table 2).

We did not observe differences in tube dependency among patients treated with adjuvant (chemo)radiotherapy versus those in the definitive treatment setting (log rank *p* = 0.65; Fig. 2b). Also, there were no differences between the control and intervention group regarding inlying gastrostomy tubes after 6 (log rank *p* = 0.1) and after 12 months (log rank *p* = 0.71).

### Association between DVH parameters and long-term PEG tube dependency

First, the influence each DVH parameter for all contoured swallowing structures (ICM, MCM, SCM, CPM, esophageal inlet, and larynx) was studied separately based on the treatment setting (definitive vs. adjuvant). Regarding long-term tube dependence after 6 months, no DVH parameter could be identified which was associated with long-term PEG use. After 12 months, the parameter ICM V65Gy (%) showed a *p*-value of 0.04 in the definitive treatment setting (Mann–Whitney U‑test; *p* = 0.038). Here, the median volume (%) to the ICM receiving at least 65 Gy equaled 81 (25.1–99.1)% as compared to 23.1 (0–85.3)% in gastrostomy tube-free patients.

Second, the univariable analysis was performed on all patients after prophylactic or reactive PEG tube placement (regardless of the therapy setting): The DVH parameter larynx V50Gy (percentage [%] of laryngeal volume) was predictive for long-term PEG use after 6 months (*p* = 0.041). Even after 12 months, larynx V50Gy (%) was still associated with long-term tube dependence (*p* = 0.042). Other parameters with impact on long-term PEG use after 12 months were ICM V60Gy (%) and ICM V65Gy (%) (*p* = 0.047 and *p* = 0.042, respectively; Table 2). Neither at 6 nor at 12 months after therapy completion was GTV found to be an influencing factor.

**Fig. 3:**
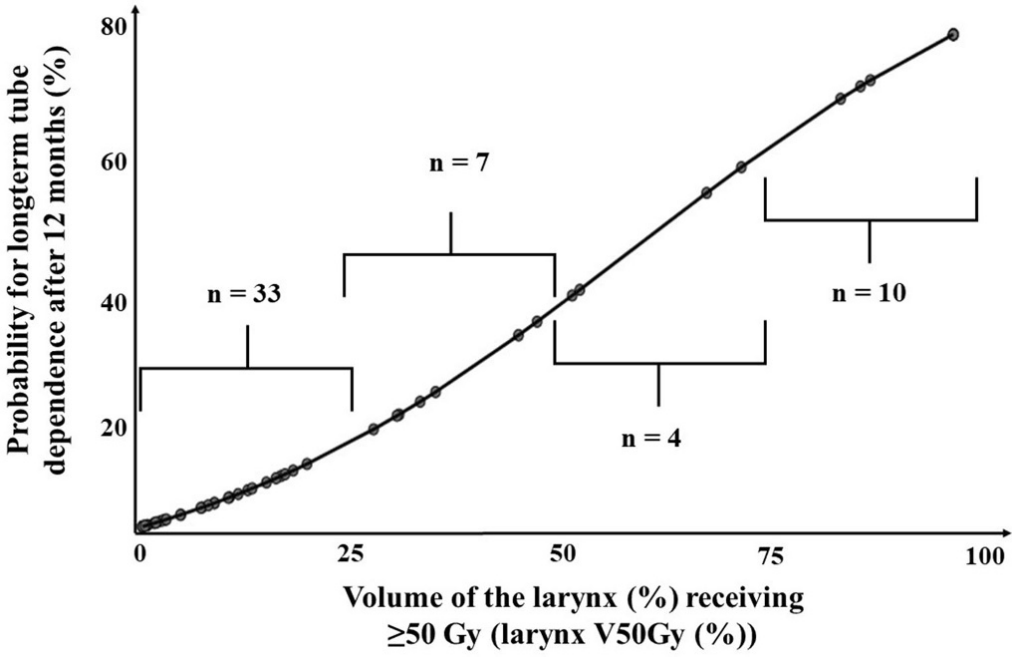
Dose–response relationship between the parameter larynx V50Gy (%) and the probability for long-term gastrostomy tube dependence after 12 months. Patient numbers lying within the respective intervals are indicated (not available for 7 patients). *VxGy (%) *organ volume (%) that received ≥ x Gy

Finally, all identified relevant DVH parameters for tube dependence after 12 months (larynx V50Gy [%], ICM V60Gy [%] and ICM V65Gy [%]) were entered into a multivariable regression model: Only larynx V50Gy (%) was identified as an independent predictor (Table 2). The dose–response relationship between the DVH parameter larynx V50Gy (%) and the probability for long-term gastrostomy tube dependence after 12 months is shown in Fig. 3.

By means of ROC analysis, the cut-off value of 53% was derived for the parameter larynx V50Gy (%) (> 53% vs. ≤ 53%; AUC = 0.74, 95% CI 0.54–0.89, *p* = 0.04).

## Discussion

We were able to identify the larynx as the main critical structure when talking about long-term tube dependence after (chemo)radiotherapy.

Prospective data on long-term tube feeding dependence or dysphagia and dosimetric considerations in patients after radiotherapy alone or combined chemoradiotherapy are very limited [15, 16]. The few available studies are either retrospective analyses and/or were published more than 10 years ago [1, 2, 14, 17]. According to Wopken et al., who screened approximately 2600 studies to identify prognostic factors for prolonged tube dependence at 6 months after radiotherapy, only 4 studies investigated the association between DVH parameters and tube dependence [1, 16–18]. Interestingly, these authors stated that predictive factors for prolonged gastrostomy tube feeding may include social factors like the usage of narcotics prior to radiotherapy and living alone during treatment [15, 19]. In our patient cohort, clinical factors like tumor stage, location, therapy setting (definitive vs. adjuvant), or the administration of concurrent chemotherapy were not associated with prolonged gastrostomy tube use after 12 months. According to Lango et al., postradiotherapy neck dissection increased the risk for feeding tube dependence at 18 months [20]. However, posttreatment neck dissection was not performed in our patients. Siano et al. retrospectively examined the prognostic influence of feeding tubes in 42 HNC patients. They observed a worse survival in patients with PEG tubes than in their counterparts without gastrostomy tubes. These authors concluded that the presence of PEG tubes had a negative impact on survival, whereas the reason remained hypothetical (e.g., weight loss) [21]. In accordance, we observed a negative effect of long-term PEG use on survival. Since our previous works showed that malnutrition negatively impacts survival in HNC patients [5, 8], these results are consistent [5, 21].

Caudell et al. concluded that the doses to the larynx and the pharyngeal constrictor muscles were crucial in predicting swallowing complications and, thus, requiring a gastrostomy tube after 12 months. More specifically, doses exceeding a Dmean of 41 Gy and the volume receiving 60 Gy (V60Gy) of more than 24% to larynx were predictive DVH parameters. Also, a volume of more than 12% receiving 60 Gy to the ICM was associated with prolonged PEG tube dependence. DVH parameters of the other pharyngeal constrictors (MCM: V65 > 75% and SCM: V65 > 33%) were predictive of posttherapeutic dilation-worthy strictures [1]. Li et al. [14] retrospectively examined 39 patients with HNC under concurrent chemoradiotherapy. In their cohort, ICM V65Gy > 30%, ICM V60Gy > 60%, ICM Dmean > 60 Gy, and CPM Dmax > 62 Gy were connected to long-term PEG tube dependence at > 192 days (6.4 months). One of the largest retrospective (and prospective) studies included 141 patients and was conducted by Vlacich et al. [17]. Most of their patients (62%) required a PEG tube. They concluded that the dose reduction to the ICM Dmean ≤ 41 Gy and V40Gy ≤ 41% helps in lowering the rate of long-term PEG need [17]. In accordance, Caglar et al. observed that doses given to the ICM correlated with swallowing complications like aspiration and stricture. Another predictive parameter was the laryngeal dose (below V50Gy < 21% no complications seen) [2]. Sanguineti et al. examined 171 patients with oropharyngeal cancer (radiotherapy: *n* = 58 and combined chemoradiotherapy: *n* = 113). In patients undergoing combined chemoradiotherapy, prophylactic gastrostomy tube placement was performed. The DVH parameters larynx V50Gy (%) and Dmean to the SCM were regarded independent predictors of prolonged tube dependence at 7 months. The authors attributed this effect to fibrosis of the larynx and the constrictor muscles [16]. In accordance with Sanguineti et al., larynx V50Gy (%) was associated with long-term tube feeding at 6 and 12 months in our patients. Our estimated threshold dose to the laryngeal volume receiving at least 50 Gy was set at 53%. Other authors reported on thresholds for larynx V50Gy at 41% and 92% [1, 16]. After performing multivariable analysis, ICM V60Gy (%) and ICM V65Gy (%) did not show to be independent predictors for our cohort. In comparison, Caudell at al. proposed a cut-off value for ICM V60Gy at 12%, while Li et al. suggested V60Gy > 60% being linked to prolonged gastrostomy tube dependence [1, 14]. The threshold for ICM V65Gy varied among different authors (between 6 and 30%) [1, 14].

The major limitation of this study is its small sample size, which could have masked underlying effects. Also, we had assumed that the intervention (nutritional counseling every 2 weeks during [chemo]radiotherapy) would sustainably improve nutritional status and, thus, reduce the number of long-term feeding tube dependency in the intervention group. However, we could not prove this. As discussed in our previous works, we suspect a psychological bias by our permanent study team caring for our study participants in both the control and intervention group. Overall, this could have led to a bias in the sense of an unintentional alignment between the control and intervention group. Another important limitation is that some PEG tubes may still have been in situ without being used for nutritional support, thus, creating a false sense of dependence, and affecting the interpretation of “dependency”.

In conclusion, long-term gastrostomy dependence was found to be strongly associated with an exposure of laryngeal structures (specifically, V50Gy ≥ 53%) during radiotherapy. Consequently, the avoidance of supraglottic as well as glottic structures is warranted.
